# Macrophage-mediated glucolipotoxicity via myeloid-related protein 8/toll-like receptor 4 signaling in diabetic nephropathy 

**DOI:** 10.1007/s10157-013-0922-5

**Published:** 2013-12-20

**Authors:** Takashige Kuwabara, Kiyoshi Mori, Masashi Mukoyama, Masato Kasahara, Hideki Yokoi, Kazuwa Nakao

**Affiliations:** 1 Department of Medicine and Clinical Science, Kyoto University Graduate School of Medicine, Kyoto University Hospital, Kyoto, 606-8507 Japan; 2 Medical Innovation Center, Kyoto University Graduate School of Medicine, Kyoto, 606-8507 Japan; 3 Department of EBM Research, Institute for Advancement of Clinical and Translational Science, Kyoto University Hospital, Kyoto, Japan

**Keywords:** Diabetic nephropathy, Glucolipotoxicity, Macrophage, Toll-like receptor

## Abstract

Dyslipidemia is an independent risk factor for the development and progression of diabetic nephropathy (DN). In this review, we summarize mouse models with both diabetes and dyslipidemia, and their associated complications. We then discuss molecules potentially involved in deterioration of DN by dyslipidemia. We focus especially upon toll-like receptor 4 (TLR4) and one of its endogenous ligands, myeloid-related protein 8 (MRP8 or S100A8), since we have found that their mRNA levels are commonly increased in glomeruli of type 1 (streptozotocin [STZ]-induced) and type 2 (*A-ZIP/F-1* lipoatrophic) diabetic mice. Gene expression of *MRP8* and *Tlr4* is further upregulated during worsening of STZ-induced DN by a high fat diet (HFD). Moreover, these HFD-induced changes are accompanied by enhanced gene expression of *CCAAT element binding protein β* and phosphorylation of c-Jun N-terminal kinase in the kidney, which have also been reported in pancreatic β cells under diabetic-hyperlipidemic conditions. Effects of a HFD upon DN are cancelled in *Tlr4* knockout mice. Macrophages are the predominant source of MRP8 in glomeruli. In cultured macrophages, combinatorial treatment with high glucose and palmitate amplifies *MRP8* expression in a *Tlr4*-dependent manner, and recombinant MRP8 protein markedly increases gene expression of the inflammatory cytokines *interleukin-1β* and *tumor necrosis factor α*. Here, we propose ‘macrophage-mediated glucolipotoxicity’ via activation of MRP8/TLR4 signaling as a novel mechanism of pathophysiology for DN.

## Introduction

Since only one-third of patients with type 1 diabetes develop diabetic nephropathy (DN), we should consider the role of factors other than hyperglycemia in the pathophysiology of DN, including genetic, epigenetic, environmental and metabolic aspects. Several reports describe hyperlipidemia or dyslipidemia as an independent risk factor for the progression of DN in type 1 and type 2 diabetes, as well as for atherosclerotic complications [1–4]. Using type 1 (streptozotocin [STZ]-induced) and type 2 (*db/db*) diabetic mouse models, we have confirmed that treatment of diabetic mice with a high fat diet (HFD) exacerbates albuminuria and glomerular lesions [5]. Of note, single nucleotide polymorphisms in *acetyl-CoA carboxylase β* gene, which plays an important role in the regulation of fatty acid metabolism, exhibit a potent association with proteinuria in patients with type 2 diabetes [6, 7]. Accordingly, a concept of synergistic toxicity caused by glucose and lipid, described as ‘glucolipotoxicity’, has emerged in recent years. However, the underlying molecular mechanism is still obscure, especially in renal complication [8]. Here we will discuss diabetic-hyperlipidemic mouse models and glucolipotoxicity in the kidney.

## Diabetic-hyperlipidemic mouse models

As described above, several clinical and experimental phenomena have highlighted the synergistic effects of hyperglycemia and hyperlipidemia upon the development and progression of diabetic complications including nephropathy. Despite the fact that there are several limitations associated with the difference in hyperlipidemia between rodents and humans, mouse models are still most widely used to study complications caused by diabetes and hyperlipidemia. The reasons include small animal size, short generation time, the ease of induction of diabetes, hyperlipidemia or gene manipulation, and cost effectiveness [9]. Hence, in the last decade diabetic-hyperlipidemic mouse models have been used for genetic modification, pharmacological treatment and/or some particular chow diets that abundantly contain fat and/or cholesterol. In this section, representative mouse models are summarized.

### *Apolipoprotein E*-deficient mice treated with streptozotocin (*ApoE* KO + STZ)

*ApoE* KO + STZ mice are one of the most popular diabetic-hyperlipidemic mouse models. This model shows not only hypercholesterolemia and hypertriglyceridemia, but also accelerated aortic atherosclerotic lesions [10–12] and nephropathy [13–15] associated with diabetes. These reports revealed that advanced glycation end-products [13, 14] and endoplasmic reticulum (ER) stress [16, 17] are candidate mediators of glucolipotoxicity in *ApoE* KO + STZ mice.

### *Low-density lipoprotein (LDL) receptor*-deficient mice treated with STZ (*LDLR* KO + STZ)

*LDLR* KO + STZ mice show dyslipidemia including high LDL cholesterol, low high-density lipoprotein (HDL) cholesterol levels and hypertriglyceridemia, mimicking human metabolic syndrome [18]. Moreover, addition of a HFD exacerbates hypertriglyceridemia, hypercholesterolemia, and diabetic renal lesions (including glomerular and tubulointerstitial macrophage infiltration) in this model [19]. The authors [19] referred to an earlier work indicating that irradiation-induced depletion of bone marrow cells (including monocytes) reduces renal injury in STZ-diabetic rats [20].

### STZ-induced diabetic mice with HFD feeding (STZ + HFD)

A supplemental HFD on STZ-treated diabetic mice increases blood triglyceride and free fatty acid concentrations, at least in part, because of insulin deficiency, suggesting that this model might be useful especially for analyzing pathophysiology by high triglyceride-rich lipoprotein and/or high free fatty acids coexisting with high glucose conditions. In STZ + HFD mice, there are several reports describing vascular complications such as cardiovascular dysfunction [21], retinopathy [22], neuropathy [23] and nephropathy [5, 24].

**Fig. 1:**
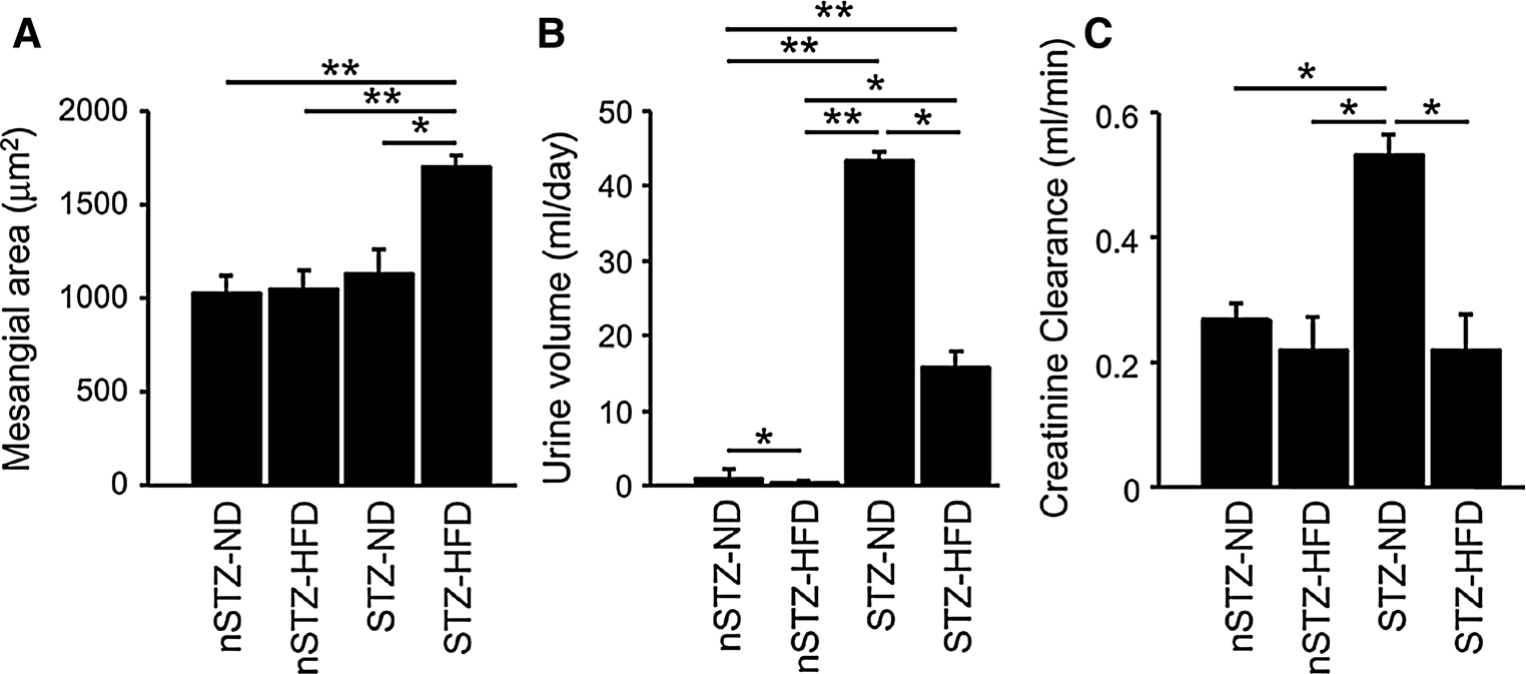
Effects of STZ and/or HFD upon mesangial expansion (**a**), urine volume (**b**) and creatinine clearance (**c**) in wild-type mice. *nSTZ-ND* non STZ-normal diet, *nSTZ-HFD* non STZ-high fat diet, *STZ-ND* STZ-normal diet, *STZ-HFD* STZ-high fat diet. Data are mean ± SEM. *n* = 4–11. *p < 0.01, **p < 0.001. Modified from Kuwabara and others [5]

Treatment of wild-type mice with STZ and HFD synergistically increases albuminuria [5] and expands mesangial area (Fig. 1). Induction of diabetes by STZ causes a marked increase in urine volume and creatinine clearance of normal diet-fed and HFD-fed animals, respectively, suggesting that glomerular hyperfiltration has occurred. On the other hand, HFD treatment reduces urine volume and creatinine clearance in STZ mice (Fig. 1), suggesting that HFD is not causing more hyperfiltration but is causing non-hemodynamic actions which will be discussed below.

### *A-ZIP/F-1* lipoatrophic diabetic mice

*A-ZIP/F-1* mice are a genetic mouse model of lipoatrophic diabetes, characterized by severe insulin resistance, dyslipidemia including hypertriglyceridemia and high free fatty acids, and fatty liver [25, 26]. This model is based upon dominant-negative expression of B-ZIP transcription factors of both C/EBP and Jun families under the control of aP2 enhancer/promoter, causing paucity of adipose tissue. *A-ZIP/F-1* mice may serve as a useful tool for studying DN, because they manifest severe nephrotic syndrome and typical histopathological renal lesions which are glomerular hypertrophy, diffuse and pronounced mesangial expansion and accumulation of extracellular matrix [27]. Notably, these renal changes are reversible to some extent by replacement therapy with a fat-derived hormone leptin [27].

### Other mouse models

There are a few other diabetic-hyperlipidemic mouse models such as non-obese diabetic mice or *Ins2* ^Akita^ diabetic mice combined with HFD feeding [28, 29], but their renal involvement has not been characterized well. Regardless of the models described above, differences in genetic backgrounds critically affect glucose and lipid metabolism among mouse strains [30]. Furthermore, even similar levels of hyperglycemia cause distinct renal changes among different strains and species. For instance, the DBA/2 strain is highly susceptible to DN, whereas the C57BL/6 strain is relatively resistant [31–33]. In addition, since cholesteryl ester transfer protein is inactive in rodents, HDL is the dominant lipoprotein in mice [34]. Apolipoprotein B in rodents also differs from that in humans [35].

## Molecules involved in glucolipotoxicity in the kidney and pancreatic β cells

Although glucotoxicity and lipotoxicity were originally proposed as independent concepts, Prentki et al. reported a novel concept of glucolipotoxicity in pancreatic β cells in 1996. They reported that elevated ambient levels of glucose and free fatty acid cause synergistic inhibition of insulin secretion [36]. On the other hand, they reported that increased intracellular glucose-derived metabolites inhibit enzymes for β-oxidation, leading to cytosolic accumulation of lipids [37]. Subsequently, there have been several reports about the molecular mechanism underlying glucolipotoxicity involved in pancreatic β cell dysfunction and insulin resistance [38–40]. Furthermore, phenomena of glucolipotoxicity are also observed in DN of humans [1–4] and rodents [41, 42], but their pathophysiology remains largely unknown [8]. Here, we will compare glucolipotoxicity upon pancreatic β cell dysfunction and DN.

### c-Jun N-terminal kinase (JNK)

**Fig. 2:**
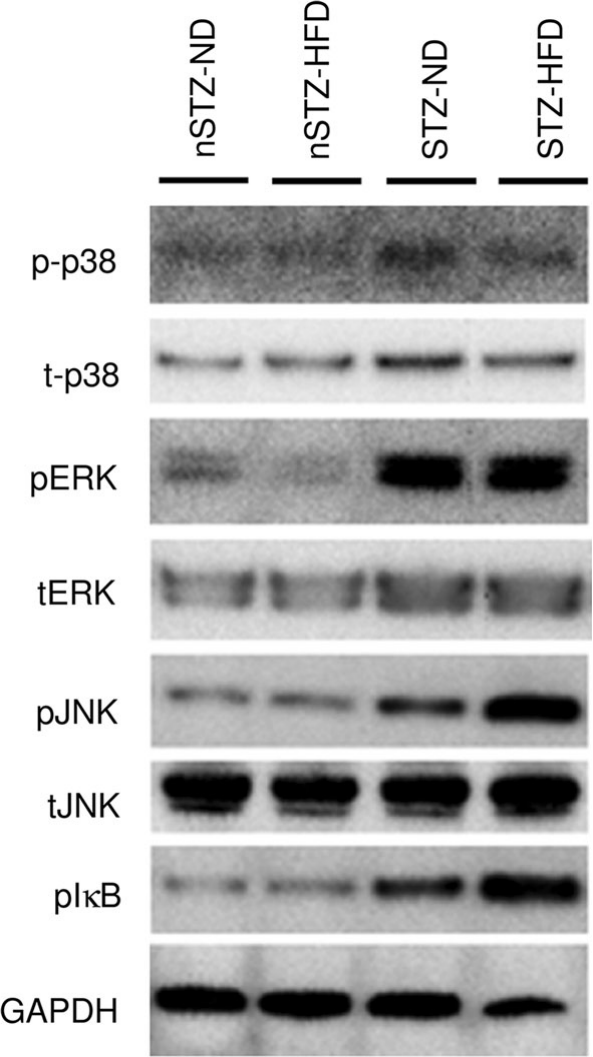
Western blot analysis for phosphorylation of MAP kinases and IκB in kidney of STZ + HFD mice. *p-/t-p38* phosphorylated/total p38 MAP kinase, *p/tERK* phosphorylated/total extracellular signal-regulated kinase, *p/tJNK* phosphorylated/total c-Jun N-terminal kinase, *pIκB* phosphorylated inhibitor of κB. Modified from Kuwabara and others [5]

JNK plays a pivotal role in ER stress-induced ‘unfolded protein response’ in innate immune system [43]. It was later revealed that ER stress-induced JNK activation is associated with chronic inflammation or high ambient fatty acid levels in obesity or type 2 diabetes [44, 45]. In pancreatic β-cells, high glucose concentrations augment lipotoxicity through JNK activation, at least partly, in an ER stress-dependent manner [46, 47]. In our diabetic-hyperlipidemic model [5], treatment with STZ and HFD synergistically increases phosphorylation of IκB and mRNA expression of pro-inflammatory genes in the kidney, in parallel with phosphorylation of JNK, but not with phosphorylation of other mitogen-activated protein (MAP) kinases such as p38 or extracellular signal-regulated kinase (ERK) (Fig. 2).

### CCAAT element binding protein beta (C/EBPβ)

CCAAT element binding protein beta (C/EBPβ) is one of the transcriptional repressors of insulin gene and induced by chronic hyperglycemia [48]. C/EBPβ is increased by fatty acids through the Per-Arnt-Sim kinase (PASK) pathway [49] in pancreatic β cells. Since PASK is also induced by high glucose conditions, these mechanisms may possibly exert glucolipotoxic effects. In the kidney, C/EBPβ is increased in diabetic rats, but not other C/EBP isoforms [50]. Furthermore, renal upregulation of *C/EBPβ* mRNA in STZ-induced diabetic mice is further enhanced by additional HFD feeding in our experiments [5].

Of note, JNK/AP-1 and C/EBPβ pathways may also contribute to glucolipotoxicity-induced renal damage through upregulation of myeloid-related protein 8 (MRP8, also known as S100A8 or calgranulin A), whose gene promoter region contains AP-1 binding site [51, 52] and C/EBP motif [53, 54], as discussed in the next section.

### Fetuin A

Over the last few years, there has been growing evidence for fatty acid-induced lipotoxicity, such as insulin resistance, through toll-like receptor 4 (TLR4) [55–57]. However, it is still controversial whether fatty acid stimulates TLR4 directly or indirectly. Recently, fetuin A has been identified as an adopter protein combining fatty acids and TLR4 [58], and its plasma levels are elevated in diabetic humans and mice [59, 60]. ER stress induced by high glucose and palmitate increases the expression of fetuin A [60], suggesting that fetuin A could hypothetically participate in glucolipotoxicity upon macrophages.

### MRP8/TLR4

**Fig. 3:**
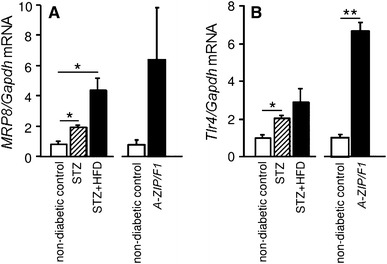
Glomerular gene expression of *MRP8 *(**a**) and *Tlr4* (**b**) in STZ + HFD and lipoatrophic *A-ZIP/F-1* mice determined by TaqMan real-time PCR. *White bars* non-diabetic control group, *striped bars* diabetic group, *black bars* diabetic-hyperlipidemic group. Data are mean ± SEM. *n* = 4–7. *p < 0.01, **p < 0.001. Modified from Kuwabara and others [5]

**Fig. 4:**
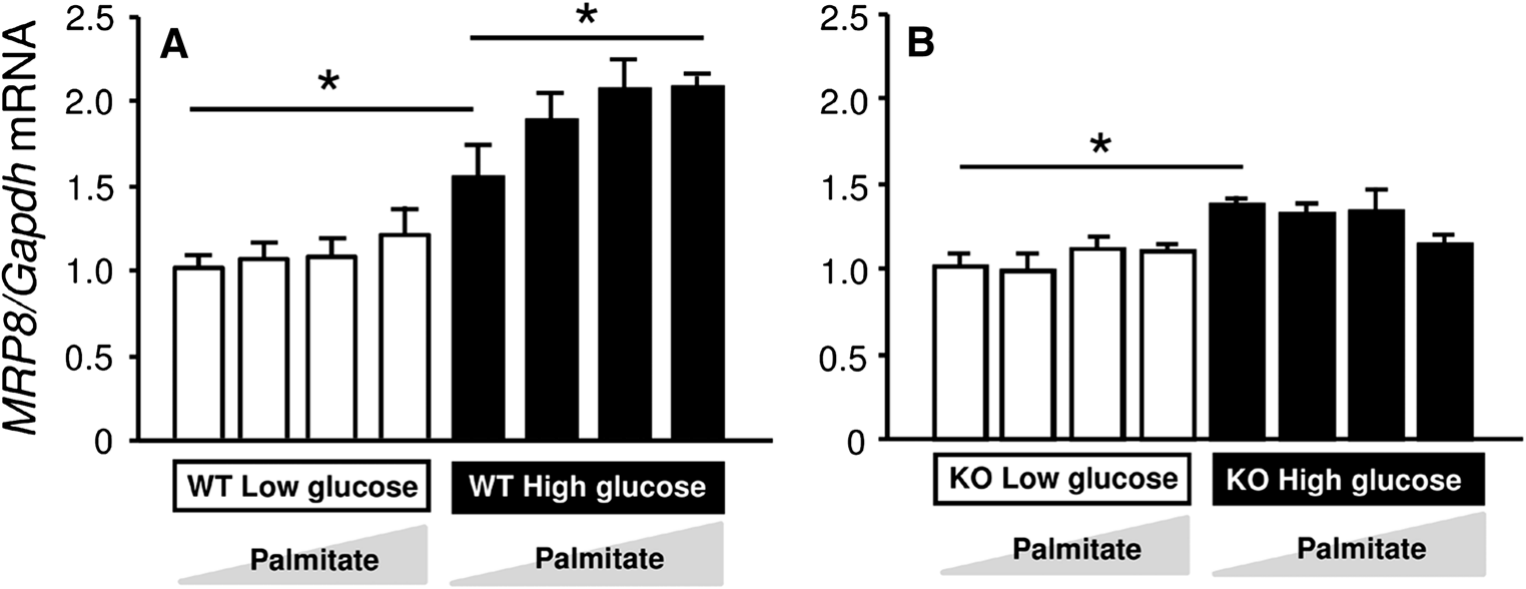
Gene expression of *MRP8* and effects of glucose or fatty acid in bone marrow-derived macrophages (BMDMs) determined by TaqMan real-time PCR. BMDMs generated from wild-type (WT, **a**) or *Tlr4* knockout (KO, **b**) mice were cultured under low-glucose (100 mg/dl, *white bars*) or high-glucose (450 mg/dl, *black bars*) conditions, and were stimulated with palmitate (0, 10, 50, and 200 μM, respectively, from the *left*) for 24 h. Data are mean ± SEM. *n* = 6. *p < 0.05. Modified from Kuwabara and others [5]

**Fig. 5:**
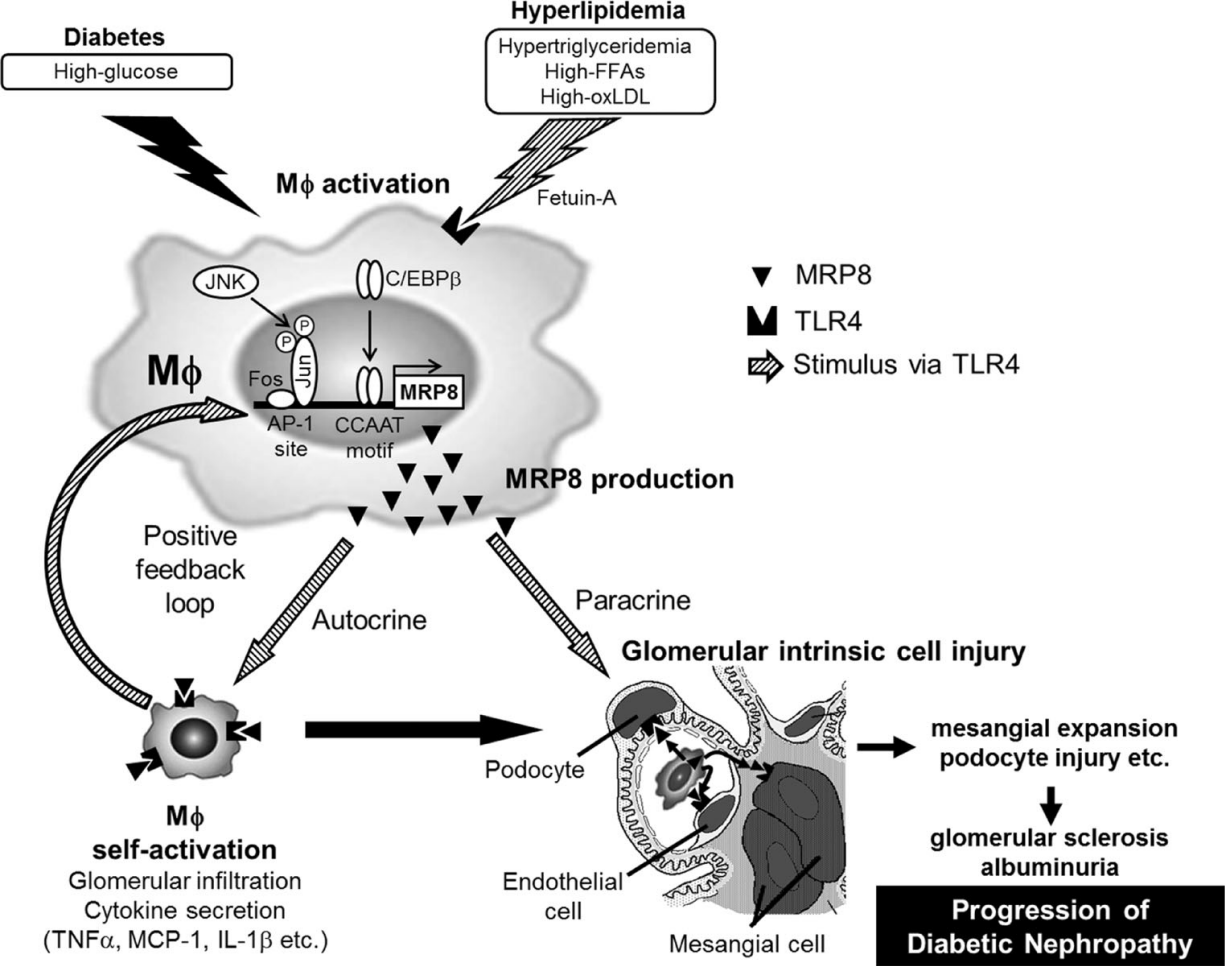
Proposed mechanism of macrophage-mediated glucolipotoxicity in diabetic nephropathy. Hyperlipidemia (or high free fatty acids) activates circulating macrophages through TLR4-mediated upregulation of MRP8, specifically under hyperglycemic conditions. These synergistic effects upon MRPã8 production in macrophages might be mediated by fetuin A and transcription factors AP-1 and CEBP/β. Macrophage activation is enhanced by a positive feedback, mediated by MRP8/TLR4 interaction in an autocrine fashion. Since glomerular intrinsic cells (such as podocytes, mesangial cells and endothelial cells) reportedly express TLR4, they can be activated through multiple pathways including (1) MRP8 from blood circulation, (2) MRP8 and inflammatory cytokines produced by glomerulus-infiltrating macrophages, and (3) hyperlipidemia. Activation of glomerular cells results in mesangial expansion and podocyte injury, further leading to glomerular sclerosis (fibrosis) and albuminuria

MRP8 was originally identified as a cytoplasmic calcium-binding protein in neutrophils and monocytes [61]. MRP8, by making a heterodimer with MRP14 (or S100A9), has become widely recognized as a potent endogenous ligand for TLR4 in various diseases including septic shock and vascular and autoimmune disorders [62–64]. To identify candidate disease-modifying molecules in DN, we have performed microarray analysis using isolated glomeruli from two different diabetic models of mice—STZ-induced insulin-dependent diabetic mice and lipoatrophic insulin-resistant A-ZIP/F-1 mice. We then focused upon *MRP8* and *Tlr4*, because expression of both genes is commonly increased in these two models [5]. It is noteworthy that diabetic-hyperlipidemic mice such as STZ-HFD mice or *A-ZIP/F-1* mice show remarkable upregulation of *MRP8* and *Tlr4* compared to control non-diabetic mice (Fig. 3). Since macrophages are identified as the major source of MRP8 in the glomeruli of STZ-HFD mice [5], we examined the effects of high glucose and fatty acid on the expression of *MRP8* (Fig. 4) and *Tlr4* in cultured macrophages. This in vitro study showed that treatment with fatty acid amplifies *MRP8* expression only under high ambient glucose conditions. Although *Tlr4* is expressed slightly more in high glucose conditions than in low glucose conditions, fatty acid does not alter *Tlr4* expression [5]. In addition, synergistic effects with high glucose and fatty acid on macrophages and diabetic kidneys are abrogated by *Tlr4* deletion [5] (Fig. 4). Moreover, we have observed that recombinant MRP8 protein markedly increases gene expression of the inflammatory cytokines *interleukin-1β* and *tumor necrosis factor α *(*TNF-α*) in cultured macrophages (submitted) [62]. Similarly, macrophages also play an important role in insulin resistance and β-cell dysfunction through fatty acid-induced TLR4 activation [65, 66]. Particularly in the kidney, MRP8 produced by infiltrated macrophages might exert glucolipotoxic effects upon diabetic glomeruli in a paracrine manner, potentially leading to mesangial expansion, podocyte injury, glomerular sclerosis and albuminuria (Fig. 5), because TLR4 is reportedly expressed in healthy or injured glomerular intrinsic cells including mesangial cells [67, 68], endothelial cells [67, 69] and podocytes [70, 71]. Taken together, we propose ‘macrophage-mediated glucolipotoxicity’ via activation of MRP8/TLR4 signaling as a novel concept for pathophysiology of DN (Fig. 5).

To understand the clinical implication of MRP8 expression in humans, we have carried out immunohistochemical analysis of MRP8 expression in renal biopsy samples from patients with DN, obesity-related glomerulopathy (ORG) and non-obese, non-diabetic controls (which are minor glomerular abnormality [MGA] and minimal change nephrotic syndrome [MCNS]). We have not been able to obtain reliable antibody against TLR4 to date. The rank orders of glomerular and tubulointerstitial MRP8 protein expression levels are DN > ORG > MCNS > MGA. Glomerular MRP8 expression is strongly correlated to the extent of proteinuria at 1 year after renal biopsy, whereas tubulointerstitial MRP8 expression is associated with worsening of renal function within a year, suggesting that renal MRP8 expression may become a new biomarker for DN (submitted).

## The role of M1 and M2 macrophages in DN with glucolipotoxicity

**Fig. 6:**
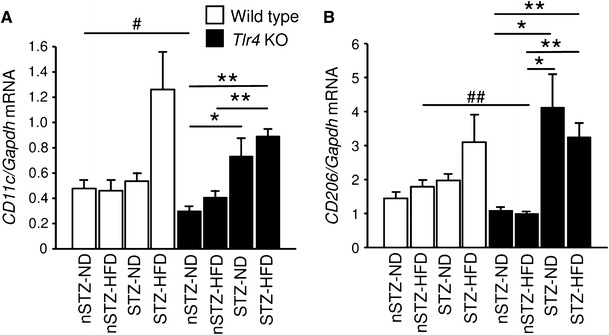
Glomerular gene expression of M1 (**a**) and M2 (**b**) macrophage markers in STZ-HFD mice determined by TaqMan real-time PCR. Data are mean ± SEM. *n* = 4–11. *p < 0.05, **p < 0.01. ^#^ *p* < 0.05, ^##^ *p* < 0.01 for similarly treated *Tlr4* KO versus wild-type

There are several subtypes of macrophages including M1 and M2 in tissue injury and repair [72–74]. During the course of renal ischemia/reperfusion injury [75] and unilateral ureteral obstruction [76], switch from proinflammatory M1 to anti-inflammatory or profibrotic M2 subtype occurs in macrophages infiltrating the tubulointerstitium. Here, we have carried out preliminary analysis of M1 and M2 macrophages in glomeruli of STZ + HFD mice by studying gene expression levels of *CD11c* (or *Itgax*) and *CD206* (or *Mrc1*) as markers of M1 and M2 subtypes, respectively [77, 78] (Fig. 6). In wild-type mice, treatment with STZ alone does not affect glomerular expression of *CD11c* and *CD206* genes, and addition of HFD to STZ causes a 100 % increase in *CD11c* and a 30 % increase in *CD206*, suggesting relative predominance of M1 subtype in diabetic-hyperlipidemic conditions. Furthermore, in *Tlr4* KO mice, the stimulatory effects of HFD upon STZ treatment are canceled both for *CD11c* and *CD206* genes, and simple STZ treatment increases *CD11c* expression by two-fold and increases *CD206* expression by three-fold, suggesting the presence of M2 predominant status. These results imply that TLR4-mediated signal is partially suppressing M2 subtype in STZ-normal diet mice and enhancing M1 subtype in STZ-HFD mice. These findings are in good agreement with previous reports indicating that treatment of macrophages with MRP8 induces M1 subtype (through TLR4 as lipopolysaccharide does) [61, 72, 76] and MRP8-expressing macrophages exhibits M1 characteristics by secretion of TNF-α and interleukin-6 [74, 79]. Formally, M1/M2 subtype analysis had to be carried out by analyzing isolated macrophages extracted from tissues.

Furthermore, in STZ + HFD animals, the levels of macrophage infiltration and extracellular matrix accumulation are proportional and progressive, suggesting that M1–M2 switching does not occur spontaneously in this model of DN. In glomeruli of STZ + HFD mice, >80 % of MRP8 signals co-localize with macrophage marker Mac2 (or Lgals3) [5], whereas collecting duct epithelial cells are the main source of MRP8 expression in unilateral ureteral obstruction [76].

In conclusion, a number of epidemiological and experimental studies have revealed that glucotoxicity and lipotoxicity cause synergistic effects upon the development and progression of DN. Macrophages have emerged as a potential contributor for mediating glucolipotoxicity through activation of MRP8/TLR4 signaling in diabetic glomeruli in our experiments. Although further studies are needed to understand regulation and potential role of MRP8/TLR4 signaling, targeting key molecules involved in this pathway may lead to novel therapeutic strategy to combat DN.
